# Targeted therapy in NPM1-mutated AML: Knowns and unknowns

**DOI:** 10.3389/fonc.2022.972606

**Published:** 2022-09-27

**Authors:** Rong Wang, Pan Xu, Lin-Lin Chang, Shi-Zhong Zhang, Hong-Hu Zhu

**Affiliations:** ^1^ Department of Hematology, the First Affiliated Hospital, School of Medicine, Zhejiang University, Hangzhou, China; ^2^ Zhejiang Province Key Laboratory of Hematology Oncology Diagnosis and Treatment, Hangzhou, China; ^3^ Department of Physiology, Medical College of China Three Gorges University, Yichang, China; ^4^ Zhejiang University Cancer Center, Hangzhou, China; ^5^ Zhejiang Laboratory for Systems & Precision Medicine, Zhejiang University Medical Center, Hangzhou, China; ^6^ Institute of Translational Medicine, Zhejiang University School of Medicine, Hangzhou, China

**Keywords:** NPM1, AML, targeted therapy, venetoclax, menin inhibitors, XPO1 inhibitors

## Abstract

Acute myeloid leukemia (AML) is a heterogeneous disease characterized by malignant proliferation of myeloid hematopoietic stem/progenitor cells. NPM1 represents the most frequently mutated gene in AML and approximately 30% of AML cases carry NPM1 mutations. Mutated NPM1 result in the cytoplasmic localization of NPM1 (NPM1c). NPM1c interacts with other proteins to block myeloid differentiation, promote cell proliferation and impair DNA damage repair. NPM1 is a good prognostic marker, but some patients ultimately relapse or fail to respond to therapy. It is urgent for us to find optimal therapies for NPM1-mutated AML. Efficacy of multiple drugs is under investigation in NPM1-mutated AML, and several clinical trials have been registered. In this review, we summarize the present knowledge of therapy and focus on the possible therapeutic interventions for NPM1-mutated AML.

## Introduction

Nucleophosmin (NPM1) is the most common mutated gene in acute myeloid leukemia (AML). AML with NPM1 mutations accounts for approximately 30% of adult AML, which is characterized by the cytoplasmic localization of NPM1 (NPM1c) (1). NPM1-mutated AML was recognized as a distinct entity in the World Health Organization classification of myeloid neoplasms.

NPM1, shuttling between the nucleus and cytoplasm, is predominantly located in the nucleus (2, 3). NPM1 protein contains three structural domains including N terminal, central and C terminal domain. Nuclear export signals (NESs), located in N terminal domain, promote the translocation of NPM1 from the nucleus to the cytoplasm (3, 4). Nucleolar localization signals (NoLS), formed in highly conserved aromatic region of C terminal domain, is critical for the localization of NPM1 to the nucleus (5). The nuclear export of NPM1 is mediated by the interaction of two NESs and the nuclear exporter exportin 1 (XPO1) (3). NPM1 is a multifunctional protein involved in diverse cellular functions such as ribosome synthesis, genomic stability, cellular growth and stress response (6–9).

NPM1 mutations result in the generation of a new C-terminal NES and the loss of tryptophan residues 288 and 290, which endow mutated-NPM1 stronger nuclear export ability and ultimately lead to the cytoplasmic localization of NPM1 (10, 11). NPM1c mediates cytoplasmic dislocation of promyelocytic leukemia (PML) nuclear bodies (NB) (12). Researchers found that NPM1c interacts and delocalizes PU.1, FBW7γ and APE1, which block myeloid differentiation, promote cell proliferation and impair DNA damage repair, respectively (13–15).

NPM1-mutated AML is a kind of AML with favorable prognosis. The overall survival rate was about 40% and complete remission (CR) rate was about 80% (16). However, approximately 50% of patients will eventually relapse (17). The standard therapy of NPM1-mutated AML patients includes “3+7” induction chemotherapy and consolidation therapy. NPM1 often co-exists with fms-like receptor tyrosine kinase-3 internal tandem duplication (FLT3-ITD), which results in poor survival and high relapse rates. Allogeneic hematopoietic stem cell transplantation (allo-HSCT) and FLT3 inhibitors may be considered as important choices for these high-risk patients. It should be underscored that despite the favorable outcome of NPM1-mutated AML patients, disease-free survival (DFS) and overall survival (OS) of older NPM1-mutated patients remain disappointing and worse than those in younger NPM1-mutated patients (18). This may be partly due to treatment options, disease biology and age-related factors.

**Figure 1:**
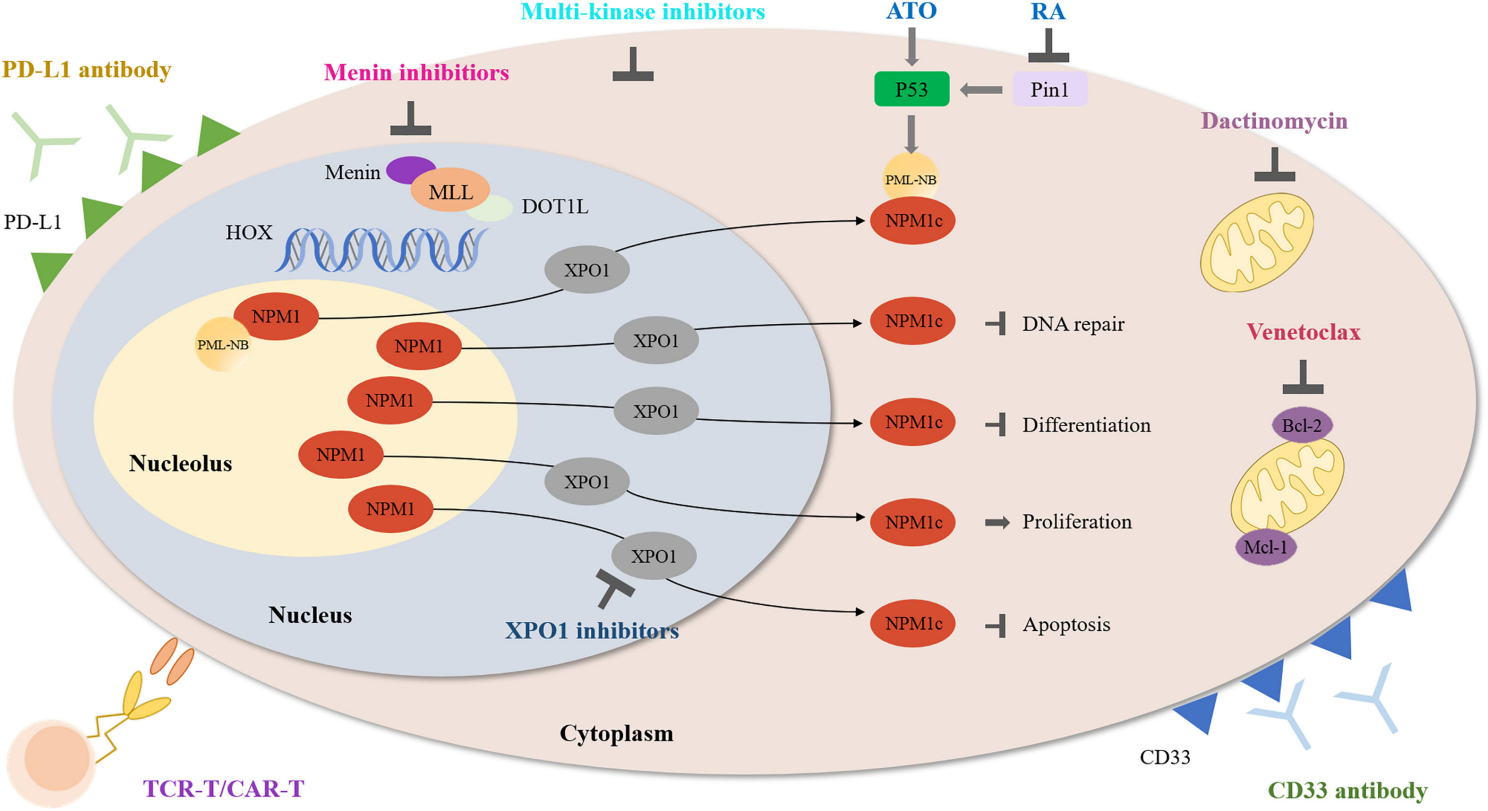
Schematic diagram describing the function of multiple agents in NPM1-mutated AML.

It has been about 15 years since NPM1-mutated AML was first discovered. However, there is no consensus over how to treat this type of AML, especially relapsed NPM1-mutated AML. Up to now, several studies targeting NPM1-mutated AML are undergoing, including inhibiting NPM1c functions, interfering with abnormal transport of NPM1c protein, promoting NPM1c degradation and immunotherapy such as monoclonal antibodies. Herein, we summarize available data (**Table 1**) and ongoing clinical trials (**Table 2**) and focus on the potential targeted therapy (**Figure 1**) of NPM1-mutated AML.

**Table 1:** Summary of venetoclax-based therapies in NPM1-mutated AML.

Basic information	Interventions	Settings	Numbers of NPM1-mutated AML patients	Clinical outcomes (CR/CRi)
**Prospective clinical studies**				
NCT02203773 (phase 1)	Venetoclax + Decitabine/Azacitidine	ND AML	N = 23	CR + CRi = 21/23 = 91.5%
NCT02287233 (phase 1/2)	Venetoclax + LDAC	ND AML	N = 9	CR + CRi = 8/9 = 89%
NCT03069352 (phase 3)	Venetoclax + LDAC	ND AML	N = 18	CR + CRi = 14/18 = 78%
NCT02993523 (phase 3)	Venetoclax + Azacitidine	ND AML	N = 27	CR + CRi = 18/27 = 66.7%
ACTRN12616000445471 (phase 1b)	Venetoclax + 5 plus 2 (cytarabine + idarubicin)	ND AML	N = 10	CR + CRi = 8/10 = 80%
NCT03214562 (phase 1b/2)	Venetoclax + FLAG+IDA	ND AML and R/R AML	N = 8	CR + CRi = 8/8 = 100%
**Real-world experience**				
2019	Venetoclax + Azacitidine	ND AML	N = 8	CR + CRi = 8/8 = 100%
2021	Venetoclax + Decitabine	R/R AML	N = 7	CR + CRi = 5/7 = 71.4%
2021	Venetoclax + HMA	R/R AML	N = 3	CR + CRi = 2/3 = 66.7%
**ASH abstracts**				
2019, NCT03586609, phase 2	Venetoclax + Cladribine +LDAC/Azacitidine	ND AML	N = 6	CR + CRi = 6/6 = 100%
2019, retrospective study	Venetoclax + Decitabine/Azacitidine/LDAC/Mylotarg	ND AML and R/R AML	N = 2	CR + CRi = 2/2 = 100%
2020, real-world outcomes	Venetoclax + HMA/LDAC/IC	ND AML and R/R AML	N = 7	CR + CRi = 6/7 = 86%
2020, retrospective study	Venetoclax + Decitabine/Azacitidine	ND AML and R/R AML	N = 21	CR + CRi = 18/21 = 86%
2020, retrospective study	Venetoclax + HMA	ND AML and R/R AML	N = 18	CR + CRi = 16/18 = 88.9%
2021, R/R AML patients with NPM1 mutation	Venetoclax + IC (cytarabine/idarubicin ± nucleoside analog) or Venentoclax + LIC (HMA/LDAC)	R/R AML	N = 12	CR + CRi = 10/12 = 83%
2021, NCT03404193, phase 2	Venetoclax + Decitabine	ND AML and R/R AML	N = 47	CR + CRi = 36/47 = 76.6%
2021, retrospective study	Venetoclax + Azacitidine	ND AML and R/R AML	N = 18	CR + CRi = 14/18 = 77.8%
**Summary**				
			N =244	CR + CRi = 200/244 = 82%

ND, newly diagnosed. LDAC, low-dose cytarabine. FLAG+IDA, fludarabine, cytarabine, granulocyte colony-stimulating factor, and idarubicin. HMA, hypomethylating agents. IC, intensive chemotherapy (cytarabine/idarubicin ± nucleoside analog). LIC, low intensity chemotherapy (HMA/LDAC).

**Table 2:** Summary of ongoing or completed clinical trials in NPM1-mutated AML.

Clinical trials identifier	Trial phase	Status	Intervention	Comments
NCT00893399	3	Completed	Gemtuzumab Ozogamicin (Mylotarg) Standard chemotherapy (Idarubicin, Etoposide, Cytarabine, ATRA, Pegfilgrastim)	Evaluating efficacy
NCT01237808	3	Completed	Cytarabine, Etoposide, All-trans retinoic acid	Evaluating efficacy
NCT03031249	1/2	Recruiting	Cytarabine, All-trans retinoic acid, Arsenic Trioxide	Evaluating safety and efficacy
NCT03769532	2	Recruiting	Pembrolizumab, Azacitidine	Evaluating safety and efficacy
NCT04689815	2	Recruiting	Oral Arsenic Trioxide Formulation	Evaluating efficacy
NCT04867928	2	Recruiting	Venetoclax, Azacitidine	Evaluating efficacy
NCT05020665	3	Recruiting	Entospletinib, Placebo, Cytarabine, Anthracycline	Evaluating efficacy
NCT04067336	1/2	Recruiting	KO-539	Two NPM1-mutated AML patients obtained CR
NCT04065399	1/2	Recruiting	SNDX-5613, Cobicistat	The overall response rate of NPM1-mutated AML: 38% (5/13)
NCT04811560	1	Recruiting	JNJ-75276617	Evaluating safety and tolerability of JNJ-75276617
NCT04988555	1/2	Recruiting	DSP-5336	Evaluating safety, tolerability and clinical activity of DSP-5336
NCT04752163	1/2	Recruiting	DS-1594b, Azacitidine, Venetoclax	Evaluating safety, toxicity and efficacy of DS-1594b
2014-000693-18	2	Completed	Dactinomycin	Evaluating anti-tumor activity and safety
2014-003490-41	2	Recruiting	Dactinomycin	Evaluating anti-tumor activity and safety

## Venetoclax-based therapies

B-cell lymphoma 2 (Bcl-2), an anti-apoptotic protein, is overexpressed in AML. High Bcl-2 expression is associated with survival of AML cells and chemotherapy resistance (19, 20). Venetoclax is a potent and selective small molecule inhibitor of Bcl-2, has shown efficacy in preclinical and clinical practice (21, 22). Recently, safety and efficacy of venetoclax-based therapies, in combination with hypomethylating agents (HMAs) or low-dose cytarabine (LDAC), has been confirmed in several AML clinical studies. To figure out the effect of venetoclax-based therapies on NPM1-mutated AML, we summarize the data from recent prospective clinical studies, real-world reports and the latest ASH abstracts (**Table 1**).

### Venetoclax + HMA/LDAC/IC

The phase 1 clinical trial of venetoclax with decitabine or azacitidine (NCT02203773) enrolled 145 AML patients and NPM1-mutated AML accounts for 16% (N = 23) (23). CR and CR with incomplete count recovery (CRi) (CR + CRi) was observed in 21 NPM1-mutated patients. In the phase 3 clinical trial of venetoclax plus azacitidine, 66.7% of NPM1-mutated AML patients achieved CR + CRi (NCT02993523) (24). The phase 1/2, phase 3 clinical trials of venetoclax and LDAC were successively conducted in AML patients (NCT02287233, NCT03069352). Patients with NPM1 mutations represented 11% and 9% of the study cohort and experienced CR + CRi at 89% and 78%, respectively (25, 26). In the clinical trials of venetoclax in combination with intensive chemotherapy (IC), patients with NPM1 mutations also had good responses. NPM1-mutated AML patients attained CR and CRi at 80% and 100% in the venetoclax combined with 5 + 2 (cytarabine + idarubicin) study and venetoclax combined with FLAG + IDA (fludarabine, cytarabine, granulocyte colony-stimulating factor, and idarubicin) study, respectively (ACTRN12616000445471, NCT03214562) (27, 28).

In real-world settings, venetoclax combined with HMA also gained good outcomes in NPM1-mutated AML patients. CR and CRi were achieved at 100%, 71.4% and 66.7% in three reports, respectively (29–31). We also collect venetoclax-based regimens data from the latest ASH abstracts, CR and CRi rates were high in NPM1-mutated AML patients ranging from 76.6% to 100% (32–39).

Furthermore, venetoclax was recently identified as a selective agent for NPM1-mutated AML through clinical drugs screening (40). A retrospective analysis compared outcomes of NPM1-mutated AML patients treated with 3 approaches including venetoclax plus HMA, HMA and intensive chemotherapy (IC). This analysis demonstrated that venetoclax plus HMAs significantly reduced the risk of death and achieved a higher CR rate when compared with standard IC or HMAs (41). Venetoclax plus LDAC showed encouraging activity in eradicating persistent or relapsing mutated NPM1 measurable residual disease (MRD) (42). The multicenter and prospective clinical trials of venetoclax-based regimens are required to confirm its safety and efficacy in NPM1-mutated AML. A phase 2, multicenter trial evaluating the efficacy of venetoclax plus azacitidine in molecular relapse/progression has been registered (NCT04867928).

### Venetoclax + ATO

In addition to the most common combinations between venetoclax and HMA, LDAC or IC, there are several new combinations under investigation, such as the ones with arsenic trioxide (ATO). ATO, as an ancient drug, has exerted its function in several malignancies. Both ATO and venetoclax can downregulate Bcl-2 expression to induce apoptosis (43). Myeloid cell leukemia sequence 1 (MCL-1) is critical for the survival of AML cells and plays an essential role in venetoclax resistance (44, 45). ATO was reported to attenuate MCL-1 upregulation induce by venetoclax (46). The synergistic antileukemic activity of ATO and venetoclax was also confirmed in primary leukemia stem cells from AML patients (46). Therefore, this combination might represent an alternative option for NPM1-mutated AML. ATO and venetoclax synergistically induces the apoptosis of NPM1-mutated OCI-AML3 cells *in vitro* and showed anti-leukemia activity in two relapsed and/or refractory (R/R) NPM1-mutated AML patients (47).

The aforesaid results highlight the promising efficacy of venetoclax-based regimens, providing a rationale for further trials in NPM1-mutated AML. Current venetoclax-based regimens are mainly applied in elderly patients who are unfit for chemotherapy or young patients who are ineligible for standard induction therapy. More studies are required to expand the application of this approach, for example, to achieve greater overall survival in young patients. Furthermore, future researches should concentrate on optimizing the venetoclax-based therapies and overcoming venetoclax resistance.

## Menin inhibitors

NPM1-mutated AML cells are characterized by high expression of HOXA and HOXB clusters, which are necessary for the maintenance of the leukemic state (48). Histone modifiers MLL1 and DOT1L control HOX and FLT3 expression and differentiation in NPM1-mutated AML (49). Combinatorial inhibition of menin-MLL1 and DOT1L showed synergistic activity against primary AML cells in this study. Another preclinical result also indicated that inhibition of menin-MLL1 reversed leukemic development of NPM1-mutated AML mice models (50). It was reported that menin-MLL1 inhibition combined with venetoclax demonstrated anti-leukemia activity in primary NPM1-mutated AML samples (51). It seems that targeting menin could be a therapeutic strategy in NPM1-mutated AML.

FLT3-ITD often co-exists with mutated NPM1, accounting for approximately 40% of NPM1-mutated AML. Combining menin inhibitors with FLT3 inhibitors induced synergistic inhibition of proliferation and enhanced apoptosis in AML blasts (52). The combination of menin and FLT3 inhibitors significantly reduced leukemia burden and induced the long-term remissions in a PDX model with both NPM1 and FLT3-ITD mutations (53). Since XPO1 inhibition potently downregulate HOX expression in NPM1-mutated AML, the combination of menin and XPO1 inhibitors appeals as a rational therapeutic option in NPM1-mutated AML (48).

Several clinical studies are recruiting to assess the safety and efficacy of menin inhibitors such as SNDX-5613 and KO-539 on leukemia with MLL-rearrangement or NPM1 mutation (NCT04067336, NCT04065399, NCT04811560, NCT04752163, **Table 2**). Early results demonstrated tolerance and biologic activity of KO-539 (54). This phase 1/2A study evaluated clinical activity in 6 R/R AML patients and KO-539 induced CR in two patients. One patient achieved MRD-negative CR, who had AML with NPM1, DNMT3A, and KMT2D mutations and received KO-539 at 200 mg daily as the eighth line of treatment. SNDX-5613 exhibited safety and promising antileukemic activity in R/R MLL-rearrangement and NPM1-mutated AML in preliminary results (55). As of data cutoff on October 18, 2021, the overall response rate in 13 NPM1-mutated AML patients was 38%. The most common side effects included prolonged QTc, nausea, vomiting and differentiation syndrome.

## XPO1 inhibitors

Exportin 1 (XPO1) is a nuclear exporter implicated in the export of proteins and RNAs (56). NPM1 mutation results in the increased nuclear export ability of mutated NPM1 (10, 11). XPO1 inhibitors can relocate mutated NPM1 to the nucleus. However, XPO1 inhibitors are not NPM1-specific and also inhibit nuclear export of other proteins such as TP53 and P21.

Considering the relationship between XPO1 and NPM1, XPO1 inhibitors might be a promising approach for NPM1-mutated AML. The combination of selinexor and venetoclax showed a synergistic effect on the anti-leukemic activity of AML cells (57). Current studies mainly focus on the effects of XPO1 inhibitors in AML, not specifically in NPM1-mutated AML. To date, the combinations of selinexor and traditional chemotherapy, such as decitabine, cytarabine, mitoxantrone and idarubicin, are under study (58–60). However, systemic toxicities of selinexor, such as nausea and anorexia, limit its clinical usage to twice per week. Eltanexor, a second-generation XPO1 inhibitor, exhibits lower blood-brain penetration, improved tolerability and better anti-leukemic efficacy when compared with selinexor (61, 62). The combination of eltanexor and venetoclax reduce cell viability and induce apoptosis of AML cell lines (63). This combination therapy also enhanced anti-leukemia effect in AML cell-derived and patient-derived xenograft models. Eltanexor seems to be a prospective drug and further investigations are needed to validate the clinical activity in NPM1-mutated AML.

XPO1 is widely expressed in normal cells and interacts with hundreds of proteins, inhibition of XPO1 might generate some side-effects such as hematologic adverse events. Future efforts should focus on combining XPO1 inhibitors with either traditional chemotherapy or novel agents to enhance efficacy and safety.

## ATO plus ATRA

Arsenic trioxide (ATO) plus all-trans retinoic acid (ATRA) had been proved a successful strategy in acute promyelocytic leukemia (APL), a unique AML subtype characterized by the fusion protein of promyelocytic leukemia (PML)–retinoic acid receptor ɑ (RARɑ). The combination has been proved to induce the degradation of PML-RARɑ fusion protein to cure APL (64).

NPM1-mutated AML cells are more sensitive to ATO because the presence of C-terminal cysteine 288 of NPM1c protein makes cells sensitize to oxidative stress induced by ATO (65). The anti-leukemia ability of ATO and ATRA support the further application in NPM1-mutated AML. Thus, two groups simultaneously demonstrated that the combination of ATO and ATRA induced the degradation of mutated NPM1 protein and apoptosis in both NPM1-mutated AML cell lines and primary cells (66, 67). Furthermore, ATO plus ATRA activated p53 signaling and restored nuclear organization of PML bodies. The combined treatment also significantly reduced bone marrow blasts in 3 NPM1-mutated AML patients and recovered the abnormal localization of both NPM1 and PML (67). ATRA was reported to induce mutated NPM1 degradation through the Pin1/PML/P53 axis, thereby promoting the response of blasts to chemotherapy or ATO (68). It is reported that ATRA improved survival of elderly NPM1-mutated AML patients without FLT3-ITD mutations when added to traditional chemotherapy (69). These findings provide convincing evidence for further clinical application of ATO and ATRA in NPM1-mutated AML. Relevant research are undergoing in NPM1-mutated AML (NCT03031249, NCT04689815, **Table 1**).

## Dactinomycin

Dactinomycin, a famous antibiotic, exhibits potent antibacterial and anticancer activity by inhibiting topoisomerases and RNA polymerases (70). Investigators found that low dose dactinomycin can efficiently generate stress response in NPM1-mutated cells, illustrating NPM1-mutated AML might be sensitive to nucleolar stress (71). Dactinomycin targets mitochondria particularly primed by mutant NPM1, induces ROS production and restore PML NBs formation. Dactinomycin was initially shown its efficacy on a NPM1-mutated AML patient without FLT3-ITD mutations. The patient achieved morphologic and immunohistochemical CR after two cycles of therapy and showed a molecular CR after the fourth cycle (72). The clinical safety and efficacy of dactinomycin in AML patients with NPM1 mutations was further established (71, 73). Dual targeting of mitochondria with dactinomycin and venetoclax exerts strong anti-leukemic activity in NPM1-mutated AML (74). Dactinomycin seems to be a potential clinical choice for NPM1-mutated AML. Two clinical trials have been registered to evaluate anti-leukemic activity and safety of dactinomycin in NPM1-mutated AML (**Table 1**).

## Immunotherapy

### CD33 antibody

CD33 is a myeloid differentiation antigen expressed at the very early stages of myeloid cell development and is absent outside the hematopoietic system or on pluripotent hematopoietic stem cells (75). CD33 expression was found in leukemic blasts in almost all AML patients and associated with adverse disease features (76, 77). Gemtuzumab ozogamicin (GO) is CD33-directed immunoconjugate by delivering a DNA-damaging calicheamicin derivative to exert its function.

CD33 expression was significantly higher in the NPM1-mutated AML cases compared with the NPM1-unmutated cases (78). The results support the therapeutic application of CD33 antibodies in NPM1-mutated AML. A study showed that the addition of GO to standard chemotherapy improves the event-free survival (EFS) and OS in *de novo* AML patients aged 50–70 years (79). Among this cohort, NPM1-mutated AML patients accounted for 33% of all cases.

One clinical study was registered to evaluate the efficacy of GO in NPM1-mutated AML (NCT00893399). The study failed to show significant benefits on EFS when GO was added to intensive therapy, which might be due to a higher early mortality in the GO arm. However, patients who achieved CR + CRi after induction therapy significantly had fewer relapses in the GO arm than in the standard arm (80). In the following attempts, the combinations of GO and other treatments are required to be optimized in NPM1-mutated AML.

### PD-1 and PD-L1 antibody

Programmed cell-death protein PD-1 and its ligands PD-L1 are immune checkpoint molecules that are involved in T-cell activation and dampen T-cell anti-tumor response. PD-1/PD-L1 pathway plays an essential role in tumor immune evasion, thus promoting the progression of tumor (81).

NPM1-mutated AML patients have a stronger cytotoxic T-lymphocyte response against mutated NPM1-derived peptides compared with healthy volunteers (82). Immune responses might be a contributing factor for the better prognosis of NPM1-mutated patients (83). High PD-L1 expression was detected in NPM1-mutated AML patients and predicted worse overall survival (84, 85). It should be noted that NPM1 was identified as a transcriptional regulator of PD-L1 and is associated with poor prognosis in triple-negative breast cancer (86). The aforementioned results indicated that PD-L1 might be a potential therapeutic target in NPM1-mutated AML. Unfortunately, current study suggested clinical activity of PD-L1 antibody in AML is limited (87, 88). Thus, more fundamental research and clinical studies are needed to investigate the exact role of PD-L1 in NPM1-mutated AML.

Hypomethylating agents, such as azactidine, have a dual effect against tumor immunity. In addition to enhancing anti-tumor immune response, HMAs can restrain immune response by upregulating PD-1 and PD-L1 expression, which can promote the exhaustion of tumor-specific T cells (89). It seems necessary to combine HMAs with immune checkpoint inhibitors such as PD-1 or PD-L1 antibody (90). Recently, a clinical study to evaluate the safety and efficacy of pembrolizumab when administered in combination with azacitidine in NPM1-mutated AML patients with molecular relapse was recruiting (NCT03769532, **Table 1**).

### CAR-T/TCR-T cell therapy

The adoptive immunotherapy, such as T cell receptor (TCR) and chimeric antigen receptor (CAR) T cell therapy, is an important milestone in the development of genetically modified cell therapies for leukemia. Due to low antigen expression in healthy tissues, TCR-T and CAR-T targeting tumor-associated antigens could be accompanied by severe toxicity. Neoantigens are derived from tumor-specific gene mutations but most neoantigens are encoded by patient-specific passenger mutations, which can be lost due to immunoediting and ultimately result in immune evasion (91). Nevertheless, neoantigens from driver gene mutations are unlikely to induce immune evasion because leukemic cells need to express the driver gene to maintain their malignant phenotype (92). Therefore, neoantigens derived from driver gene mutations are ideal targets for immunotherapy.

Mutated NPM1 is an essential driver gene and occurs in approximately 30% of AML. Besides the primary genetic lesion, NPM1 mutations also cooperate with other mutations to contribute to leukemogenesis (93). Moreover, NPM1-mutated protein does not exist in normal tissues, so it is an ideal leukemic-specific antigen and a potential target for NPM1-mutated AML. Recently, TCR-T and CAR-T directed against NPM1-mutated peptides obtained preliminary success in NPM1-mutated AML.

Van der Lee et al. transduced CD8+ and CD4+ T cells with the TCR for NPM1-mutated peptide, which demonstrated efficient specificity against NPM1-mutated and HLA-A2-restricted primary leukemic blasts (94). T cells transduced with TCR for NPM1-mutated protein could efficiently kill AML cells and prolonged OS of NSG mice engrafted with HLA-A*02:01-positive NPM1-mutated OCI-AML3 human cells. NPM1-mutated CAR-T cells showed efficient and specific anti-leukemia activity against NPM1c+HLA-A2+ leukemia cells and primary AML blasts (95). CAR-T cells could significantly reduce leukemia burden and prolonged survival of NSG mice engrafted with OCI-AML3 cells. Both TCR-T and CAR-T exhibit strong specificity and cytotoxicity against NPM1-muated AML without evident side effects. Further studies are warranted to investigate in clinical application and overcome potential drawbacks.

## Conclusion

NPM1-mutated AML is a clinically heterogeneous group because it almost always exists in the context of other mutations. NPM1 mutations often co-occur with FLT3, DNMT3A or other mutations to contribute to leukemogenesis (96, 97). The latest report classified NPM1-mutated AML into two novel subtypes, primitive and committed subtype, based on a stem cell signature through RNA-seq (98). Interestingly, they found that leukemic cells in the primitive subtype are more sensitive to certain kinase inhibitors. The addition of kinase inhibitors to the treatment might achieve therapeutic benefits in this specific subtype of NPM1-mutated AML. These results may prompt us to make a more accurate risk stratification of NPM1-mutated AML based on multidisciplinary technology, thereby giving a guidance for clinical treatment. Furthermore, some controversial issues in diagnosis and treatments of NPM1-mutated AML still exists. Falini et al. recently summarized how he diagnose and treat NPM1-mutated AML and he constructively proposed that NPM1 mutational status, the timing of HSCT, MRD monitoring and ELN genetic-based risk stratification should be considered during the therapy (99).

NPM1 mutations are ideal targets for MRD monitoring because they are AML-specific, frequent, stable at relapse and do not drive clonal hematopoiesis of indeterminate potential. Investigators found that MRD, as determined by real-time quantitative PCR (RT-qPCR) of NPM1-mutated transcripts, provides important prognostic information for AML (100). Patients with persistence of NPM1-mutated transcripts in blood after the second cycle of chemotherapy was associated with a greater risk of relapse and a lower rate of survival compared with those without such transcripts. In multivariate analysis, the presence of MRD was the only significant prognostic factor for relapse and death. RT-qPCR remains the standard method for MRD monitoring in NPM1-mutated AML, the application of highly sensitive digital droplet PCR and NGS will be expanded in the future.

Considering the above findings, the combination of multiple agents is the dominant trend in the future treatment of NPM1-mutated AML, such as venetoclax-based regimens and XPO1 inhibitors combinations. The pathogenesis of NPM1-mutated AML and diverse drugs combinations need to be further studied. Joint efforts should be made to overcome the limitation of currently promising drugs, such as resistance for venetoclax and toxicity for XPO1 inhibitors. Novel targeted drugs for NPM1-mutated AML are also urgently developed. We are looking forward to acquiring the consensus on treatment of NPM1-mutated AML.

## Author contributions

H-HZ and S-ZZ conceived the idea of the paper. RW, PX, and L-LC contributed equally to data collection and wrote the manuscript. All authors contributed to the article and approved the submitted version.

## Funding

Leading Innovative and Entrepreneur Team Introduction Programme of Zhejiang (2020R01006 and 2019R01001) and the “Pioneer” R&D program of Zhejiang (2022C03005).

## Conflict of interest

The authors declare that the research was conducted in the absence of any commercial or financial relationships that could be construed as a potential conflict of interest.

## Publisher’s note

All claims expressed in this article are solely those of the authors and do not necessarily represent those of their affiliated organizations, or those of the publisher, the editors and the reviewers. Any product that may be evaluated in this article, or claim that may be made by its manufacturer, is not guaranteed or endorsed by the publisher.
